# Investigating the Potential Role of Genetic and Epigenetic Variation of DNA Methyltransferase Genes in Hyperplastic Polyposis Syndrome

**DOI:** 10.1371/journal.pone.0016831

**Published:** 2011-02-10

**Authors:** Musa Drini, Nicholas C. Wong, Hamish S. Scott, Jeffrey M. Craig, Alexander Dobrovic, Chelsee A. Hewitt, Christofer Dow, Joanne P. Young, Mark A. Jenkins, Richard Saffery, Finlay A. Macrae

**Affiliations:** 1 Colorectal Medicine and Genetics, Royal Melbourne Hospital, Department of Medicine, University of Melbourne, Parkville, Victoria, Australia; 2 Gastroenterology and Hepatology Department, Canberra Hospital, Garran, Australian Capital Territory, Australia; 3 Developmental Epigenetics, Murdoch Childrens Research Institute, Department of Paediatrics, University of Melbourne, Royal Children's Hospital, Parkville, Victoria, Australia; 4 Institute of Medical and Veterinary Science, Adelaide, Australia; 5 Department of Pathology, Peter MacCallum Cancer Centre, East Melbourne, Victoria, Australia; 6 Department of Pathology, University of Melbourne, Parkville, Victoria, Australia; 7 Anatomical Pathology, Royal Melbourne Hospital, Parkville, Victoria, Australia; 8 Queensland Institute of Medical Research, Herston, Queensland, Australia; 9 MEGA Epidemiology, School of Population Health, University of Melbourne, Carlton, Victoria, Australia; Sanford-Burnham Medical Research Institute, United States of America

## Abstract

**Background:**

Hyperplastic Polyposis Syndrome (HPS) is a condition associated with multiple serrated polyps, and an increased risk of colorectal cancer (CRC). At least half of CRCs arising in HPS show a CpG island methylator phenotype (CIMP), potentially linked to aberrant DNA methyltransferase (DNMT) activity. CIMP is associated with methylation of tumor suppressor genes including regulators of DNA mismatch repair (such as *MLH1*, *MGMT*), and negative regulators of Wnt signaling (such as *WIF1*). In this study, we investigated the potential for interaction of genetic and epigenetic variation in *DNMT* genes, in the aetiology of HPS.

**Methods:**

We utilized high resolution melting (HRM) analysis to screen 45 cases with HPS for novel sequence variants in *DNMT1*, *DNMT3A*, *DNMT3B*, and *DNMT3L*. 21 polyps from 13 patients were screened for *BRAF* and *KRAS* mutations, with assessment of promoter methylation in the *DNMT1*, *DNMT3A*, *DNMT3B*, *DNMT3L MLH1, MGMT*, and *WIF1* gene promoters.

**Results:**

No pathologic germline mutations were observed in any DNA-methyltransferase gene. However, the T allele of rs62106244 (intron 10 of *DNMT1* gene) was over-represented in cases with HPS (p<0.01) compared with population controls. The *DNMT1*, *DNMT3A* and *DNMT3B* promoters were unmethylated in all instances. Interestingly, the *DNMT3L* promoter showed low levels of methylation in polyps and normal colonic mucosa relative to matched disease free cells with methylation level negatively correlated to expression level in normal colonic tissue. *DNMT3*L promoter hypomethylation was more often found in polyps harbouring *KRAS* mutations (p = 0.0053). *BRAF* mutations were common (11 out of 21 polyps), whilst *KRAS* mutations were identified in 4 of 21 polyps.

**Conclusions:**

Genetic or epigenetic alterations in *DNMT* genes do not appear to be associated with HPS, but further investigation of genetic variation at rs62106244 is justified given the high frequency of the minor allele in this case series.

## Introduction

Hyperplastic Polyposis Syndrome (HPS) is a colorectal cancer (CRC) predisposition associated with the development of multiple serrated polyps, and is defined by the World Health Organization as:

(1) at least five serrated polyps proximal to the sigmoid colon with two or more of these being >10 mm; or

(2) any number of serrated polyps proximal to the sigmoid colon in an individual who has a first-degree relative with serrated polyposis; or

(3) >20 serrated polyps of any size, but distributed throughout the colon. The implied meaning of this last criterion is that the polyps are not all present in the rectum [1].

HPS is the genetic disease model for the *serrated neoplasia pathway* of CRC development [2]–[4]. This new distinct pathway of CRC is characterized by activating mutation of the *BRAF* proto-oncogene (specifically V600E) and widespread and concordant gene promoter hypermethylation (CpG Island methylation Phenotype or CIMP) [5]–[8], and is responsible for silencing of many genes by CpG island methylation in specific cancer subtypes [9]. CIMP positive CRC cancers have distinct clinico-pathological features including proximal location, mucinous histopathology, female preponderance, and a high frequency of *BRAF* mutation [7], [10]–[12]. DNA methylation disturbances are also a feature of HPS with a large proportion of *BRAF* mutation positive HPS polyps being CIMP positive [13], [14]. Increased DNA methylation in 14 markers (*MINTs 1,2* and *31*, *p16*, *MGMT*, *MLH1, RASSF1*, *RASSF2, NORE1, RKIP, MST1, DAPK, FAS* and *CHFR*) in small hyperplastic polyps and in normal mucosa of patients with HPS suggests that there may be a genetic basis for this observation [6].

The aberrant DNA methylation found in the CIMP phenotype may be a consequence of a dysfunction in the machinery involved in establishing and maintaining normal DNA methylation [15]. DNA methyltransferases (DNMTs) are a family of proteins responsible for transfer of methyl groups specifically to cytosine in CpG dinucleotides of DNA [16], [17]. Four DNMTs (*DNMT1* [18], *DNMT3A*, *DNMT3B* [19]–[21], and *DNMT3L* [22], [23]) have been extensively characterized in mammals. *DNMT1* acts primarily on hemi-methylated double stranded DNA following DNA replication to faithfully maintain methylation patterns in daughter cells [24], [25]. *DNMT3A* and -*3B* are involved in de novo methylation, establishing tissue-specific DNA methylation marks during development [26], [27]. *DNMT3L* lacks the methyltransferase catalytic domain [28] and is thought to facilitate the action of other DNMTs by enhancing their targeting to specific loci for DNA methylation [29].

Dysregulation of DNMTs has been linked to many cancers [30], and may play a role in the CIMP phenotype in CRC [31]. Indeed recent evidence suggests that overexpression of *DNMT3B* in particular shows a significant association with CIMP high CRC [32], [33]. Given the observation that even apparently normal mucosa in patients with HPS is highly methylated [6], we investigated the potential for mutation or epigenetic disruption of *DNMT1* (CCDS12228.1), *DNMT3A* (CCDS1718.1), *DNMT3B* (CCDS13204.1) and *DNMT3L* (CCDS13705.1) in the development of HPS.

## Materials and Methods

### Patient Selection

The Melbourne Health Human Research Ethics Committee approved the study (HREC2007.081) and all participants provided written informed consent to take part. We utilized the clinical database of The Royal Melbourne Hospital Bowel Cancer Surveillance Service and the Familial Cancer Clinic, to identify patients satisfying the diagnostic criteria for HPS. Detailed patient demographics, polyp number, size, location, pedigree, family and personal history of CRC and histopathology were prospectively collected (Table 1 and Table 2).

**Table 1:** Patients' demographics.

Number of cases with HPS	45
Median age years (range)	61 (28–82)
Female	21
Median age at diagnosis	49 (26–78)
Median number of HP per case	37.5
Number of patients with HPs >10 mm	18
Patients with 5 or more coexisting adenomas	13
Number of cases who had partial/total colectomy for CRC/HPS	11/5
Number of families with HPS	2
Number of patients with FHx of CRC (FDR)	20
Personal history of CRC	11
Median age of patients with CRC	47.5

Thirty-six cases from this cohort have been previously reported [66]. CRC- colorectal cancer, FDR-first degree relative.

**Table 2:** Clinico-pathological characteristics of cases: DNMT promoter methylation analysis and *BRAF/KRAS* mutation.

*Cases*	*HPs location*	*Classification of polyps*	*Size*	*BRAF/KRAS mutation*
1	Sigmoid colon	SSA	10 mm	KRAS
2	Descending colon	SSA	8 mm	WT
3	Rectum	MVHP	5 mm	KRAS
	Transverse colon	MVHP	6 mm	KRAS
	Transverse colon	MVHP	7 mm	WT
4	Sigmoid colon	MVHP-SSA	9 mm	BRAF
	Sigmoid colon	MVHP	5 mm	BRAF
5	Transverse colon	MVHP	6 mm	BRAF
	Splenic flexure	MVHP	5 mm	BRAF
6	Descending colon	HP	9 mm	BRAF
7	Ascending colon	SSA	7 mm	BRAF
	Transverse colon	Mixed HP/SSA	5 mm	KRAS
	Descending colon	SSA	9 mm	BRAF
8	Rectum	SSA	5 mm	BRAF
9	Ascending colon	SSA	12 mm	WT
10	Rectum	SSA	4 mm	BRAF
11	Transverse colon	MixedHP/SSA	6 mm	WT
12	Transverse colon	SSA	8 mm	BRAF
	Transverse colon	SSA	5 mm	WT
13	Rectum	SSA	9 mm	BRAF
	Sigmoid colon	HP	5 mm	WT

SSA-sessile serrated polyp; HP-typical hyperplastic polyp; MVHP- microvesicular hyperplastic polyp.

DNA sequence data on this paper have been previously reported on the GenBank database.

Polyps were reviewed by a histopathologist with an interest in the HPS (CD). The use of the term *serrated polyp* in this report encompasses polyps with serrated architecture and includes micro-vesicular hyperplastic polyps, and sessile serrated polyps with or without dysplasia. Traditional serrated adenoma and adenoma were not considered in this report. We classified lesions as advanced polyps if the size of the lesion was 10 mm or more.

Control colon tissue was obtained during colonoscopy from patients who presented with abdominal pain for investigation and completed endoscopic examination was normal.

Peripheral blood was collected for germline mutation analysis and immortalized EBV transformed lymphoblast cell lines (LCLs) were generated. If surveillance colonoscopy was performed, polyp tissue was collected and stored in RNALater (Ambion-Applied Biosystems).

### Total DNA extraction

LCLs (lymphoblast cell lines) were available from 45 patients with HPS. DNA was extracted from all cases using DNeasy 96 Blood and Tissue kits according to the manufacturer's instructions (Qiagen, Hilden, Germany). In addition, DNA was extracted from 21 polyps obtained from 13 different HPS cases. DNA concentration and quality was assessed by absorbance spectrophotometry and agarose gel electrophoresis.

### RNA Isolation and Real-Time Reverse-Transcription PCR

Total RNA was isolated using Trizol Reagent (Invitrogen, Carlsbad, CA, USA) according to the manufacturer's instructions, followed by treatment with Turbo DNA-free (Ambion/Applied Biosystems, Austin, TX, USA) to remove contaminating genomic DNA. Reverse transcription using random hexamers was performed with SuperScript VILO cDNA Synthesis Kit (Invitrogen). To determine expression levels of genes of interest, quantitative RT-PCR using SYBR Green-based analysis and Master Mix (Invitrogen) was carried out with 10 µM sense and antisense primers (primers on request). Reactions were performed in triplicate and analysed using an ABI 7300 Sequence Detection System (Applied Biosystems). Relative expression levels were determined using the standard ΔCt method with GAPDH housekeeping gene used for normalisation.

### Germline mutation detection

We employed High Resolution Melting (HRM) analysis as a novel and cost effective method to search for the presence of unknown variants in DNMT genes, from 45 cases with available genomic DNA. DNA was first amplified by real time PCR in the presence of LightCycler® 480 High Resolution Melting Dye using a LightCycler® 480 System (Roche Applied Sciences). After the PCR, the successive melting curve experiment was performed in the same apparatus. Gene scanning software was used to identify sequence variation by melt profiling. PCR and DNA melting were performed in triplicate. PCR was optimized with titration of MgCl_2_ and variation in annealing temperatures. In brief, 10 ng genomic DNA was used in the PCR, Master mix (Roche Applied Sciences), and final forward and reverse primer (concentration 0.2 µM). Sense and antisense PCR primers for *DNMT* genes were designed using consensus coding sequences: *DNMT1* (CCDS12228.1), *DNMT3A* (CCDS1718.1), *DNMT3B* (CCDS13204.1) and *DNMT3L* (CCDS13705) [34]. When amplicon length exceeded 300 bp or nonspecific product was evident following electrophoresis on a 2% agarose gel, we designed alternative primer pairs using the Primer3 software package (http://biotools.umassmed.edu/bioapps/primer3_www.cgi). The online POLAND program was used to confirm that amplicons contained a single melting domain [35].

### Somatic mutation detection for *BRAF* and *KRAS*

High resolution melting (HRM) analysis was performed using a LightCycler® 480 (Roche Diagnostics, Penzberg, Germany) in a modification of the previously published method of [36]. The reaction mixture contained: 1x PCR buffer, DNA template, 200 uM dNTPs (Fisher Biotec Australia, Wembley, Western Australia), 5 µM SYTO® 9 (Invitrogen, Carlsbad, CA), and 0.5 U HotStarTaq polymerase (Qiagen, Germantown, MD, USA). Various concentrations of MgCl_2_ and primers (primers on request) were used specific to each assay in a 10 µl final reaction volume. PCR reactions were performed in triplicate.

### Quantitative DNA methylation analysis by MALDI-TOF MassARRAY EPITYPING and bisulphite sequencing

DNA was extracted from 21 polyps (13 cases with HPS) and 5 controls (colonic mucosa from non-HPS cases) using the DNeasy kit (Qiagen). Bisulphite conversion was performed using the MethylEasy Xceed kit according to manufacturer's instructions (Human Genetic Signatures, Sydney, Australia) 1–2 µg of genomic DNA was used for bisulphite conversion. The converted DNA was eluted to a final concentration of 20 ng/µl. 20 ng of converted DNA was used for PCR amplification.

We employed SEQUENOM EpiTYPER analysis [37] for detection and quantitation of DNA methylation of the promoter regions of *DNMT1*, *DNMT3A*, *DNMT3B* and *DNMT3L*. In brief, genomic DNA was bisulphite treated using MethylEasy Xceed kit (Human Genetic Signatures), PCR amplification was performed using primers directed to the promoter regions of the *DNMT* genes (primers on request). Amplicons were then subjected to the EpiTYPER chemistry. Briefly, amplicons were treated with shrimp alkaline phosphatase treatment followed by *in vitro* transcription and base specific cleavage (SEQUENOM, San Diego, CA). Samples were then analysed by MALDI-TOF mass spectroscopy and the methylation ratios obtained using EpiTYPER v1.0.5 software (SEQUENOM). Further analysis and cleaning of data was performed using the R statistical package (http://www.cran.org) and scripts developed in house. This included identification and removal of CpG units that overlapped with other peaks following mass spectroscopy. Further data cleaning and curation was then performed in R to remove CpG units that did not yield data in at least 70% of samples. Samples, from which less than 40% of CpG units yielded data, were also not included in subsequent analyses.

The *DNMT3L* amplicons were subjected to cloning and DNA sequencing as outlined in [38]. Amplicons were cloned into pGEMT-Easy Vector (Promega, Madison, WI, USA) and then transformed into DH5-α *E. coli*. Positive clones were selected for automated DNA sequencing performed by the Australian Genome Research Facility (AGRF, Melbourne, Australia). Sequencing data was then analysed using BiQ-Analyzer [39].

### Statistical analysis

After data cleaning and curation of SEQUENOM assays, data was analysed using the Heatmap.2 function in R whereby unsupervised hierarchal clustering of the samples and CpG units was performed. Dendrograms and associated heatmaps were generated according to the DNA methylation ratio of specific CpG units and samples analysed. Geometric means methylation levels were calculated for each tissue type, prior to statistical analysis using a Student's t-test.

A box and whisker plot was generated in R to display the distribution of DNA methylation ratios between polyp and germline tissues across the *DNMT3L* promoter. Calculations were performed to estimate odds ratios (ORs) and 95% confidence intervals (95% CIs) for the associations between allele frequency and disease. All statistical tests were two-sided and P-value, 0.05 was considered as a significant level of statistical evidence to reject the null hypothesis. All statistical analyses were done using Stata 10.0.

## Results

### DNA methyltransferase exon scanning by High Resolution Melting (HRM) Analysis

In order to ascertain whether the previously reported methylation disruption associated with HPS may be due to genetic variability in DNMTs, we screened a total of 92 *DNMT* exons for novel mutations by HRM analysis in LCL genomic DNA from HPS subjects. This comprised 40 exons from *DNMT1*, 17 from *DNMT3A*, 24 from *DNMT3B* and 11 from *DNMT3L.* We found three previously identified single nucleotide polymorphisms (SNPs) in *DNMT1* (synonymous rs721186 and rs2228613) and intronic (rs62106244) and one SNP in *DNMT3A* (synonymous rs2276598) (Table 3).

**Table 3:** SNPs identified with HRM on germline DNA.

Gene	SNP ID	Exon/Intron	DNA change	Clinical association
*DNMT1*	rs721186	Exon	C/T	Unknown
*DNMT1*	rs62106244	Intron	C/T	Unknown
*DNMT1*	rs2228613	Exon	A/C	Unknown
*DNMT3A*	rs2276598	Exon	C/T	Unknown

**Figure 1:**
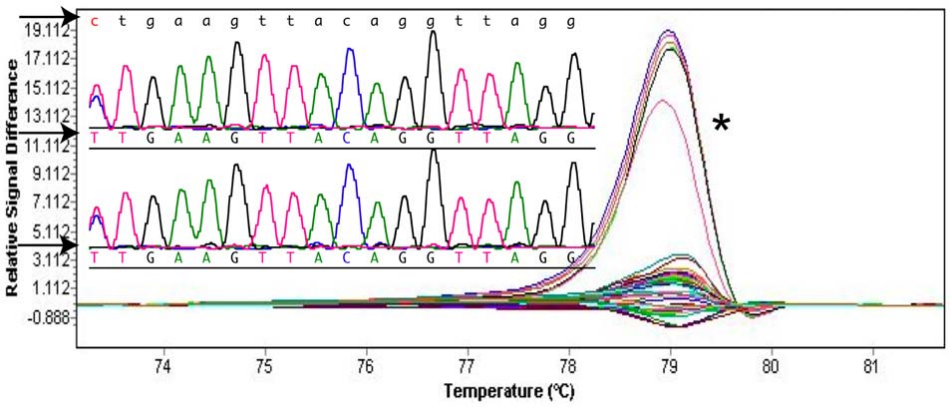
Normalized and temp-shifted difference plot for *DNMT1* gene SNP rs62106244. *Seven cases were heterozygous for rs62106244. The top arrow shows the sequence of the *DNMT1* gene.

The T allele of the rs62106244 C>T variant (Fig. 1) was present in 7 out of 45 cases all of whom were heterozygous. To establish the frequency of this uncharacterised variant in the Caucasian population generally, we scanned 300 control samples, and found 16 that were heterozygous at this site. Chi-square analysis confirmed a statistically significant over representation of both the CT genotype and T allele in HPS cases (χ^2^ = 6.66, p = 0.01; χ^2^ = 7.45, p<0.01 respectively). We observed that cases with HPS were approximately three times more likely to carry the variant (15.6%) compared to controls (5.3%), and this difference was statistically significant (p = 0.01). However, given the rarity of the variant the confidence intervals were wide (relative risk  = 2.9; 95% confidence interval 1.1 to 6.5). *DNMT3B* and *DNMT3L* exon scanning did not reveal any SNPs in our HPS cases.

### Quantitative methylation analysis of CRC CIMP-associated genes and *DNMT*s

Previous studies using a small number of patients with HPS and methylation specific PCR (MSP) have identified elevated levels of specific promoter methylation in both polyps and normal mucosa relative to non-diseased mucosa from subjects with sporadic serrated polyps [6]. This includes methylation of the *MGMT* and *MLH1* genes. In this study, we measured DNA methylation of the *IGF2* differentially methylated region (DMR), *H19, MGMT, MLH1* and *WIF1* promoters. Contrary to previous reports [40]; [13], [14] we found that, *MGMT* and *MLH1* were unmethylated (Fig. S1) in both polyps and matched normal mucosa, while there was no significant difference in DNA methylation of *IGF2, H19*, and *WIF1* (Fig. S1) between polyp and disease free tissue.

In addition to examining CIMP markers, we also examined the potential for methylation based dysregulation of the *DNMT* genes *themselves.* Little data is available regarding methylation status of this family of genes in non-diseased somatic tissue, and only limited studies have examined this in disease [41], [42]. We studied DNA methylation of the promoter region of the *DNMT1*, *-3A* and *-3B* genes in HPS cases, comparing polyp-derived DNA, normal mucosa from the same patient (where available), and mucosa from controls with no disease. All three promoter regions were generally unmethylated in both polyp and normal mucosal tissue, with less than 10% methylation detected by SEQUENOM EpiTYPER (data not shown). We also found no evidence of promoter methylation of these genes in LCL genomic DNA (data not shown).

**Figure 2:**
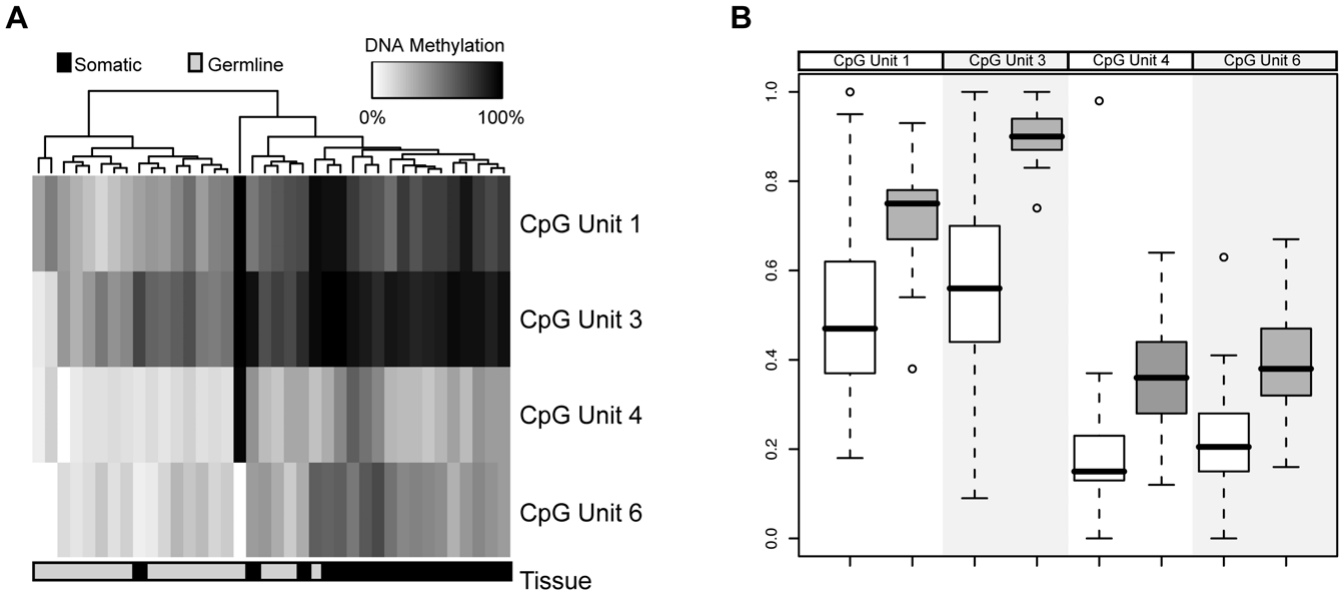
Unsupervised hierarchical clustering of the level of methylation of DNA from LCLs and somatic DNA in HPS cases for CpG_1 (analytic unit 1), CpG_6.7 (unit 2), CpG_8 (unit 3) and CpG_10 (unit 6) of the *DNMT3L* promoter region (A); Box and whisker plot comparing quantitative methylation between polyp tissue (white boxes) and peripheral blood (grey boxes) for each CpG site (B).

**Figure 3:**
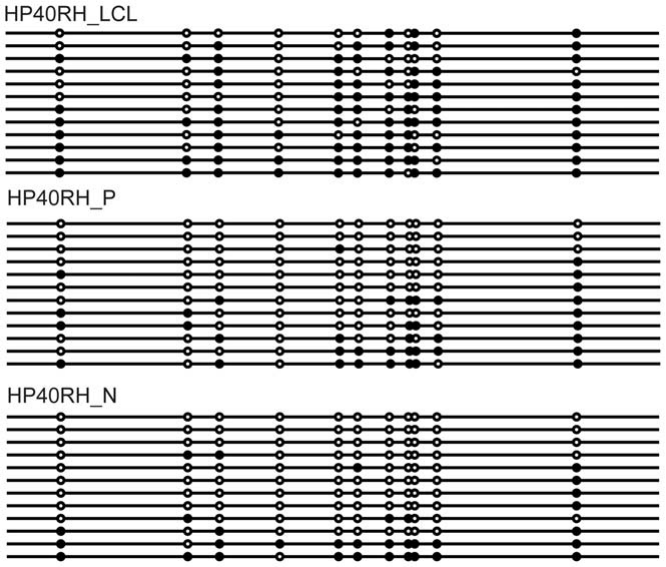
*DNMT3L* bisulphite sequencing: HP40RH_LCL lymphoblastic cell line DNA, HP40RH_P polyp DNA and normal mucosa DNA HP40RH_N. Each line represents a sequenced clone and DNA methylation information is presented as a circle denoting the CpG site where methylation could occur. Black circles represent methylated CpG's while white circles represent unmethylated CpG's. The promoter of *DNMT3L* was observed to be less methylated in normal mucosa and polyp tissue but methylated in lymphoblastic cell line DNA.

**Figure 4:**
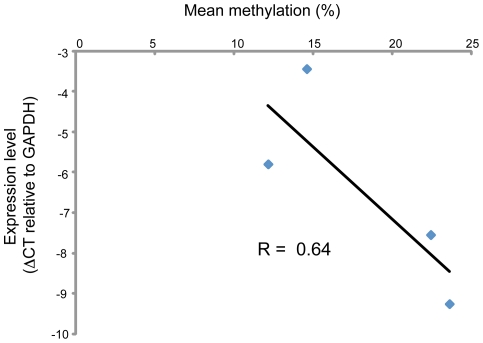
Correlation between mean methylation of *DNMT3L* promoter and gene expression level in control mucosa tissue. Mean was calculated from the combined methylation values for CpG units, 1, 2, 3 and 6.

Interestingly, we found that *DNMT3L* was *hypomethylated* in both normal gut mucosa (mean methylation 0.33, SD 0.24, n = 12) and polyp tissue (mean methylation 0.36, SD 0.25 n = 21) relative to matched LCL DNA (mean 0.57, SD 0.24 n = 19); see unsupervised clustering figure 2A and box and whisker plot figure 2B). This difference was highly significant (p = 5.1×10^−9^ Student's t-test) and was confirmed by bisulphite sequencing (Fig. 3). We were unable to test expression level directly in HPS samples due to a lack of RNA, however a negative correlation between mean methylation and expression level was found in normal colonic tissue (R =  −0.64; Fig. 4).

### *KRAS* mutation may correlate with DNMT3L promoter methylation

CIMP and *BRAF* mutation are hallmarks of serrated tumours and *BRAF* V600E has been proposed as an important biological marker for HPS specific cancers [43]. We performed *BRAF* (V600E) and *KRAS* screening with HRM on DNA from serrated polyps derived from HPS cases and controls. Consistent with previous published data [7], [44] we found that the *BRAF* V600E mutation was common in serrated polyps (11 out of 21) whereas the *KRAS* mutation was evident in only 4 out of 21. *KRAS* mutations were found primarily in polyps located in the transverse colon (two) and rectum (two). Interestingly one patient had two polyps with the *BRAF* somatic mutation and one with the *KRAS* somatic mutation, all within the right colon from the same patient. Examination of the level of *DNMT3L* promoter methylation and *KRAS* mutation-containing polyps revealed an association between these distinct genetic and epigenetic modifications (Table 4).

**Table 4:** Quantitative methylation (%) of DNMT gene promoters: 21 serrated polyps stratified by BRAF and KRAS mutation.

	Mean *DNMT1* methylation (%)	Mean *DNMT3A*methylation (%)	Mean *DNMT3B*methylation (%)	Mean *DNMT3L*methylation (%)
Polyps with BRAF mutation	3.3∧	4.3∧	21∧	37*
Polyps with*KRAS* mutation	2.8∧	4.1∧	27∧	55**
Polyps WT for BRAF and *KRAS*	3.6	4.4	25	30

∧p-value <0.05 for DNMT1, DNMT3A and DNMT3B.

*p-value  = 0.147 (BRAF/WT).

**p-value:  = 0.0053 (*KRAS*/WT).

Polyps with *KRAS* mutations were far more likely to show *DNMT3L* promoter methylation at levels similar to those found in LCLs (p = 0.0053). In contrast, polyps with the *BRAF* mutation did not show any significant association with *DNMT3L* promoter methylation.

## Discussion

Given the significant association of HPS and associated tumours with increasing levels of aberrant promoter methylation (CIMP), including extensive methylation in the normal mucosa, and the role of DNMTs in both the establishment and maintenance of DNA methylation in humans, we examined the potential association of genetic and epigenetic disruption of DNMTs in HPS.

### Genetic variation is the *DNMT1* gene is potentially associated with HPS

Variants in *DNMT1* have been identified as risk factors for disease including systemic lupus erythematosus [45]. Genetic deficiency of *DNMT3B* causes ICF syndrome, a recessive human disorder characterised by immunodeficiency, centromere instability, and facial anomalies [46]. Variants in other DNMTs (i.e. *DNMT3L, DNMT1*) have been associated with human cancers [47]–[50]. Recently, a rare variant of *DNMT3L* was specifically associated with reduced methylation of sub-telomeric regions in humans [51]. In this study we demonstrated that HPS is not associated with germline mutations of the *DNMT3A, -3B*, or *-3L* genes. Although we identified SNPs in both *DNMT1* and *DNMT3A* in HPS cases, these were generally detected at similar frequencies in non-diseased subjects and were predicted to be non-pathogenic.

The exception was rs62106244, located in the *DNMT1* gene, that was found to have a significantly higher frequency in HPS cases in comparison with controls. This SNP is not predicted to alter *DNMT1* splicing or protein, and is not evolutionarily conserved beyond primates [52]. Corresponding intronic SNPs, in other genes, have previously been associated with many diseases, including some cancers [53]. In fact as many as 45% of all trait/disease-associated SNPs identified in genome-wide association studies are intronic [54] with the pathogenic mechanisms underlying such associations remaining largely unclear. In addition, the level of linkage disequilibrium (LD) of rs62106244 in relation to other variants (i.e. haplotype status) has not yet been determined, although the entire *DNMT1* gene lies in a large block of LD.

### Common CIMP markers are absent in most HPS lesions

Perturbation of DNA methylation and loss of gene function is well documented in MSI-H CRC [55]. However, the role of aberrant DNA methylation in *precancerous* lesions remains poorly understood despite its widespread occurrence. It has been reported that alteration of DNA methylation in hepatocellular carcinoma (HCC) evolves through precancerous lesions to multistage hepatocarcinogenesis [56]. In comparison with non-diseased tissue, human cancers generally show variations in both global DNA methylation (hypomethylation) and gene-specific (hyper- or hypo methylation) status [16], [57].

Promoter methylation of *MLH1* in MSI-H sporadic colon cancers represents a classical example of aberrant methylation leading to cancer [12]. We did not find any evidence of aberrant methylation in HPS “precancerous lesions” for this traditional CIMP marker or for the *MGMT* gene. DNA methylation at other markers (*IGF2 WIF1 and H19*) was also found to be unaffected in this group of HPS cases. *MLH1* methylation is associated with high-level MSI and this is in turn usually observed in areas of dysplasia or overt carcinoma. Similarly, methylation of *MGMT* is more likely to be seen in conjunction with *KRAS* mutation. Our findings in polyps therefore are not inconsistent with published reports.

### *DNMT3L* is specifically hypomethylated in gut mucosa and HPS

Our data from a candidate gene approach to explore epigenetic abnormalities in HPS revealed a general lack of *DNMT1*, *DNMT3A* and *DNMT3B* methylation as expected in non-diseased mucosal tissue. However, *DNMT3L*, usually silenced in somatic tissues by methylation, was found to be *hypomethylated* in both HPS polyps and normal gut mucosa relative to LCLs. There was no statistically significant difference between matched normal mucosa and polyps derived from the same cases, or between colonic mucosa from HPS cases and controls (colonic mucosa from cases with normal endoscopic findings and no history of polyps). We also found elevated methylation levels in all other somatic tissues tested including kidney, peripheral blood mononuclear cells, skeletal muscle, brain, and skin fibroblasts (data not shown), suggesting that the decreased methylation seen in gut mucosa is of functional significance. This was further supported by gene expression analysis that confirmed a inverse correlation between mean methylation and gene expression levels. In mice, methylation of the *DNMT3L* promoter has been unequivocally linked to gene silencing [58], and it has been shown that this gene is primarily expressed in thymus, testis and ovaries [23], [59]. We have now added gut mucosa to the list of tissues expressing this gene. Recently the loss of DNA methylation at the *DNMT3L* promoter was found to be a positive biomarker for cervical cancer [60]. DNMT3L over expression in cervical cancer cells increases cellular proliferation, anchorage independent growth and nuclear reprogramming of cells, all central events in tumour development [61].

### An interaction between *DNMT3L* methylation and KRAS mutation?

Previous studies that have examined the relationship between *BRAF and KRAS* mutations in a wide range of colonic polyp and cancer tissue have found a mutually exclusive distribution of these mutations [7]. *DNMT3L* methylation percentage across all CpG sites analysed for cases with *BRAF* and *KRAS* mutation revealed increased methylation of the *DNMT3L* promoter in cases with a *KRAS* mutation when compared with polyps with the normal variant (p = 0.0053), however due to small number of cases this observation need to be investigated in a larger number of polyps harbouring *KRAS* mutation. Additionally we found both *BRAF* and *KRAS* mutations in a single case (two polyps harbouring *BRAF* mutation and one polyp with *KRAS* mutation), which, though relatively rare, has been previously described [44]. Lesions harbouring *KRAS* mutations have been associated with distal location within the colorectum [62] MSI-L status [63] and unfavourable prognosis [64]. Our findings with regard to *DNMT3L* methylation and *KRAS* mutation need to be investigated further as recently aberrant promoter hypomethylation of *DNMT3L* has been linked with cervical cancer tumorigenesis [60]. The increased *DNMT3L* methylation seen in *KRAS* mutated polyps is commensurate with the lower levels of methylation generally seen in these polyps relative to those with *BRAF* mutations.

### Conclusions

To date, there is no single genetic abnormality known to underlie HPS. In addition, factors determining the transition to colon carcinoma remain unknown. There have been several reports implicating *DNMT* dysregulation in carcinogenesis [41], [65]. However, the degree to which DNMTs may contribute to the development of precancerous lesions remains poorly understood. This study represents the first investigation of the possible role of the DNMT family of genes in the development of HPS. The data generated do not exclude a functional role of DNMT dysregulation in the development of HPS and associated disorders, however no exonic germline mutations were discovered, suggesting that regulation of DNMTs may occur via alternative mechanisms in this condition. It is interesting to speculate that the observed hypomethylation of the *DNMT3L* promoter in normal gut mucosa in the HPS patients identified here, may also play a role in the aberrant *de novo* establishment of tumour suppressor methylation seen in most CRC.

## Supporting Information

Figure S1 Quantitative methylation analysis (%) with SEQUENOM: genes analysed *H19, MGMT, MLH1, WIF1, BRAF* and *KRAS*. Samples: S1, S13, S10, S11 and S26 were disease free tissue.
(TIF)
