# Supplementary figures and images for: Investigating the Potential Role of Genetic and Epigenetic Variation of DNA Methyltransferase Genes in Hyperplastic Polyposis Syndrome
Source: PLoS One. 2011 Feb 10;6(2):e16831. doi: 10.1371/journal.pone.0016831 (PMC3037390; doi:10.1371/journal.pone.0016831)

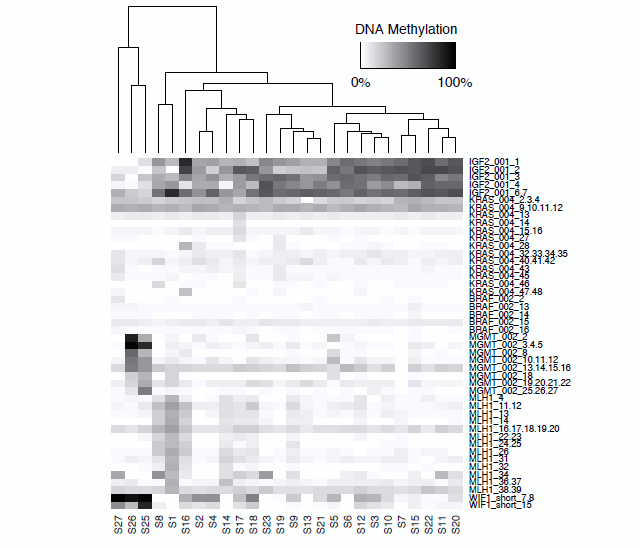

Supplement: Figure S1 — Quantitative methylation analysis (%) with SEQUENOM: genes analysed H19, MGMT, MLH1, WIF1, BRAF and KRAS. Samples: S1, S13, S10, S11 and S26 were disease free tissue. (TIF) [file pone.0016831.s001.tif]
